# Process Analytical Technology for Advanced Process Control in Biologics Manufacturing with the Aid of Macroscopic Kinetic Modeling

**DOI:** 10.3390/bioengineering5010025

**Published:** 2018-03-16

**Authors:** Martin Kornecki, Jochen Strube

**Affiliations:** Institute for Separation and Process Technology, Clausthal University of Technology, Leibnizstr. 15, 38678 Clausthal-Zellerfeld, Germany; kornecki@itv.tu-clausthal.de

**Keywords:** process analytical technology, macroscopic modeling, biologics manufacturing, upstream, downstream, turbidity, Raman, Chinese hamster ovary, process control

## Abstract

Productivity improvements of mammalian cell culture in the production of recombinant proteins have been made by optimizing cell lines, media, and process operation. This led to enhanced titers and process robustness without increasing the cost of the upstream processing (USP); however, a downstream bottleneck remains. In terms of process control improvement, the process analytical technology (PAT) initiative, initiated by the American Food and Drug Administration (FDA), aims to measure, analyze, monitor, and ultimately control all important attributes of a bioprocess. Especially, spectroscopic methods such as Raman or near-infrared spectroscopy enable one to meet these analytical requirements, preferably in-situ. In combination with chemometric techniques like partial least square (PLS) or principal component analysis (PCA), it is possible to generate soft sensors, which estimate process variables based on process and measurement models for the enhanced control of bioprocesses. Macroscopic kinetic models can be used to simulate cell metabolism. These models are able to enhance the process understanding by predicting the dynamic of cells during cultivation. In this article, in-situ turbidity (transmission, 880 nm) and ex-situ Raman spectroscopy (785 nm) measurements are combined with an offline macroscopic Monod kinetic model in order to predict substrate concentrations. Experimental data of Chinese hamster ovary cultivations in bioreactors show a sufficiently linear correlation (R^2^ ≥ 0.97) between turbidity and total cell concentration. PLS regression of Raman spectra generates a prediction model, which was validated via offline viable cell concentration measurement (RMSE ≤ 13.82, R^2^ ≥ 0.92). Based on these measurements, the macroscopic Monod model can be used to determine different process attributes, e.g., glucose concentration. In consequence, it is possible to approximately calculate (R^2^ ≥ 0.96) glucose concentration based on online cell concentration measurements using turbidity or Raman spectroscopy. Future approaches will use these online substrate concentration measurements with turbidity and Raman measurements, in combination with the kinetic model, in order to control the bioprocess in terms of feeding strategies, by employing an open platform communication (OPC) network—either in fed-batch or perfusion mode, integrated into a continuous operation of upstream and downstream.

## 1. Introduction

### 1.1. Process Analytical Technology

The steadily increasing demand for process robustness and understanding has led to the introduction of the process analytical technology (PAT) initiative in the biotechnological, biopharmaceutical, and food industry by the Food and Drug Administration (FDA) in 2004 [1,2,3]. The PAT initiative aims to measure, analyze, monitor, and ultimately control all important attributes of a bioprocess in order to maintain or improve product quality [1,4,5,6]. These attributes comprise process parameters (e.g., pH, pO_2_, gas flow) and variables (e.g., biomass/viability, substrate/metabolite/product concentration). The overall goal is to control critical process parameters that influence the cellular growth rate µ, production rates q_i_ of the product, host cell proteins, and metabolites (q_product_ q_HCP_, q_lac_, q_amm_), as well as product quality (e.g., structure, post-translational modifications, and efficacy) [7,8]. Hence, the real-time monitoring of bioreactors is crucial for an efficient, well controlled, robust bioprocess. Depending on the location of the analysis, bioreactor monitoring techniques distinguish between in-situ, which can be invasive, non-invasive, or placed in a sampling loop, or ex-situ [9]. In-situ or ex-situ spectroscopic methods are employed for the quantitative or qualitative description of process variables, due to their fast, sensitive, and reliable characteristics [9].

Exemplary methods are shown in Table 1. Spectroscopic methods aim to measure the cellular condition, as well as substrate, product, and metabolite concentration online. The common online determination of these variables using dedicated measurement devices (online glucose analyzer, cell imager) is either laborious, susceptible to maintenance efforts, or has the tendency to be used specifically for only one application. Spectroscopic probes eliminate those drawbacks by multiplexing, and process independent economic use and simple integration [4,10,11,12]. However, the analysis and interpretation of spectral data can be complex, and the correlation between responses and factors may not always be obvious [13].

Despite this, the analysis of complex spectral data, e.g., Raman, NIR, MIR, and fluorescence, can be conducted using multivariate chemometric techniques. Chemometrics comprises mathematical and statistical methods in order to design or select optimal measurement procedures and to obtain maximal information by analyzing chemical data [14]. Techniques such as Principal Component Analysis (PCA), Principal Components Regression (PCR), and Partial Least Squares Regression (PLS) are used in order to describe the correlation between responses and factors, especially in spectral data [4,9,14,15]. The application of these methods is mainly due to the non-selectivity of the spectroscopic measurements and the high collinearity of the variables [9].

**Figure 1:**
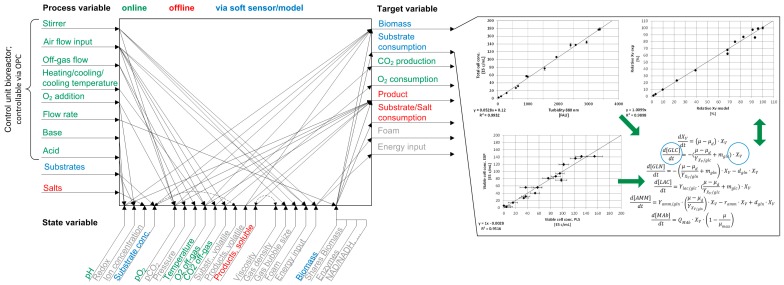
Main correlations between process, state, and target variables according to [22]. In this experimental set-up green, red, and blue variables can be measured/controlled online, offline, or via a soft sensor/kinetic model, respectively (**left**). The target variable biomass can be measured indirectly via turbidity (linear correlation) or Raman spectroscopy (PLS). The substrate consumption, e.g., glucose, can be calculated due to the relationship between cell and substrate concentration in a macroscopic model based on Monod equations (**right**).

The high resolution data acquisition of target variables such as biomass or substrates is crucial for process control and gaining deeper process understanding, due to the correlation between process parameters, the process state, and target attributes. The online, offline, and soft sensor/model determination of these variables can be seen in Figure 1. Process variables such as stirrer rate, air flow, or base addition are measured, monitored, and controlled using dedicated control units (i.e., DCU, digital control unit) or OPC (open platform communication) platforms. Variables, which are in-situ not directly accessible (e.g., biomass, substrate concentration), can be monitored employing spectroscopic techniques, for example.

Commercially available in-situ turbidity sensors, which measure the optical density (OD) of suspensions, can be implemented for the determination of total cell concentrations of mammalian cell cultures [2,16]. Inline turbidity measurement techniques distinguish between transmission, absorption, reflection, or light scattering [2,17]. The most significant advantages of turbidity probes are their measurement resolution, simple use, and implementation. However, depending on the cellular viability, the optical density can considerably differ from the viable cell concentration.

Nevertheless, turbidity probes are able to directly collect online data regarding total cell concentration and can be used for process control if the cellular viability is not decreasing (i.e., viable cell concentration does not significantly differ from total cell concentration). This makes turbidity sensors a viable monitoring and controlling technique for bioprocesses [16,18] (especially for continuous bioprocessing, e.g., perfusion turbidostat).

Therefore, techniques that provide insight into the cellular viability are favorable and should be monitored in real-time according to the ICH Q8-R2 guidance [16,19]. Raman spectroscopy, in combination with chemometric analysis methods, can, for example, be used for the determination of the total or viable cell concentration, as well as for the substrate and metabolite concentration [4,20].

The combination of turbidity and/or Raman spectroscopy with a macroscopic model such as the soft sensor enables the estimation and/or prediction of process variables, which are not directly measureable [21].

### 1.2. Macroscopic Models

Macroscopic models that predict the dynamic state of a mammalian cell culture help to gather vast information about the cellular condition, including correlations between growth and substrate uptake, as well as metabolite and product accumulation [23,24,25,26,27,28,29]. According to [23], the relationship between input (e.g., substrate concentration) and output (e.g., cell concentration) variables can be macroscopically modeled by using methods such as statistics (e.g., PCA), empirical observations (e.g., yield coefficients), and metabolic networks (e.g., metabolic flux analysis). The kinetic model can be established by neural networks and logistic or Monod-type approaches. Initiating with experimental and literature data, the correlation between input and output variables using empirical observations (i.e., yield coefficients), as well as the establishment of the kinetic using a Monod-type approach, seems to be the most straightforward method for implementation. Metabolic networks, however, represent a more detailed image of the cell [30]. However, the construction of such models is more time consuming than empirical observations, if reaction and flux rates of metabolic pathways are experimentally determined using metabolomics and not adapted from literature [31].

### 1.3. Main Metabolism of Mammalian Cells

**Figure 2:**
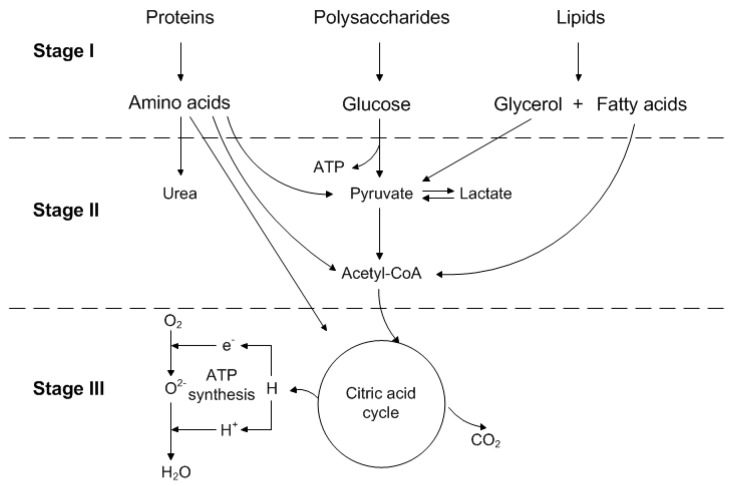
Schematic overview of the main catabolic metabolism divided into three stages, adapted from [32].

In terms of the metabolism of mammalian cells, Figure 2 shows a schematic overview of the main catabolic metabolism, which is divided into three stages [32,33,34,35]:

1. Nutrients such as polysaccharides, as well as proteins and lipids, are broken down into their components.
2. Components, derived in stage one, are converted into their common compounds, pyruvate, and acetyl-CoA.
3. Finally, acetyl-CoA is integrated into the citric acid cycle, which is accompanied by oxidative phosphorylation.

**Figure 3:**
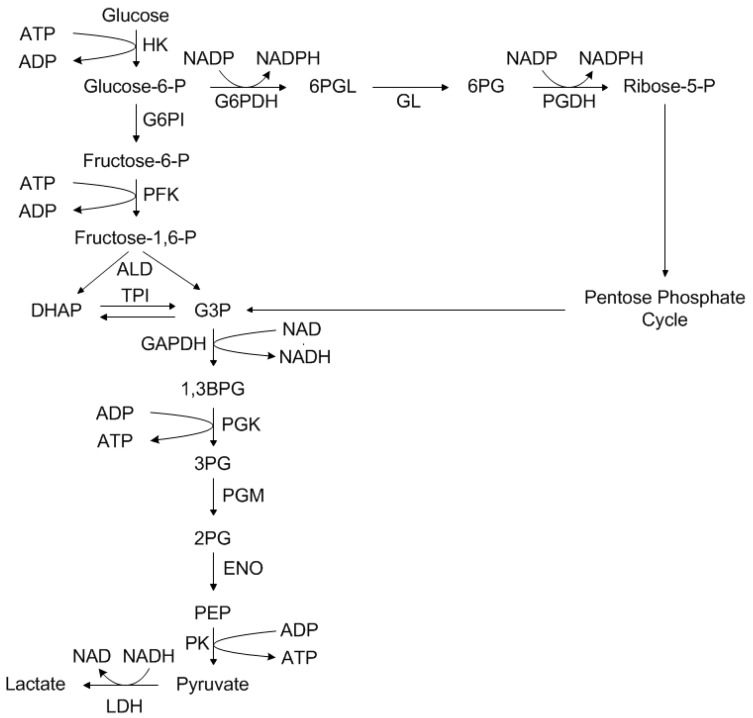
Production of lactate and pyruvate in glycolysis. HK, Hexokinase; G6PI, Glucose-6-phosphate isomerase; PFK, Phosphofructokinase; ALD, Aldolase; TPI, Triosephosphate isomerase; GAPDH, Glyceraldehyde-3-phosphate dehydrogenase; PGK, Phosphoglycerate kinase; PGM, Phosphoglycerate mutase; ENO, Enolase; PK, Pyruvate kinase; LDH, Lactate dehydrogenase; G6PDH, Glucose-6-phosphate dehydrogenase; GL, 6-Phosphogluconolactonase; PGDH, Phosphogluconate dehydrogenase; DHAP, Dihydroxyacetone phosphate; G3P, Glyceraldehyde 3-phosphate; 1,3BPG, 1,3-Bisphosphoglycerate; 3PG, 3-Phosphoglycerate; 2PG, 2-Phosphoglycerate; PEP, Phosphoenolpyruvate; 6PGL, 6-Phosphogluconolactone; 6PG, 6-Phosphogluconate.

The main glucose metabolism that initiates with glycolysis alongside the pentose phosphate cycle (synthesis of ribose 5-phosphate, a precursor of nucleotides) is depicted in Figure 3. Previous research has shown that energy production from glucose alone is not sufficient [36]. Glucose cannot, however, be added in arbitrary quantities, as excess glucose leads to substrate inhibition. This means alternative energy sources, for example the 20 proteinogenic amino acids, are required to manage increased energy requirements [37]. Due to the efficient absorption of glutamine, it is added to most cultivation media as an alternative energy source. Glutamine plays a central role in the metabolism, since it serves as a starting material in numerous reactions and syntheses. These include, for example, peptide and protein synthesis, as well as the formation of amino sugars and nucleic acid synthesis [38].

Besides the main energy sources, the resulting metabolites also play an important role in cultivation. The main by-products of glucose and glutamine metabolism are lactate and ammonium, which have a strong influence on the life span of cells and the production of the antibody [39,40,41].

In addition to pyruvate, the crucial metabolite lactate is being produced in glycolysis during the exponential growth phase. The consumption of lactate is observed during the stationary phase upon depletion of glutamine and is desirable, since it seems to correlate to improved optimal process performance [42]. However, the reduction of pH, as well as the inhibition of cell growth and production of IgG, constitutes side-effects, resulting in the increase of lactate [37]. Nevertheless, variation of pH can be reduced by an appropriate controller during a bioprocess. However, the addition of ions leads to an increased osmotic pressure, which in turn has negative effects on cell culture. At most, non-controlled cultivations in shaking flasks may be exposed to pH variation. For these reasons, the lactate content should be kept as low as possible.

**Figure 4:**
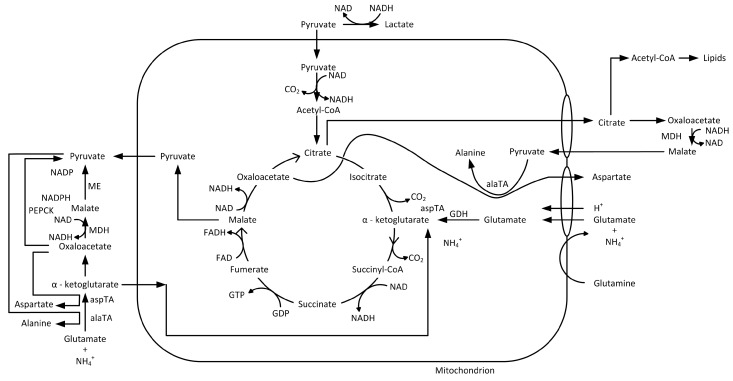
Citrate cycle, which represents the entry point into the energy metabolism, adapted from [37].

The also critical by-product ammonium (NH_4_^+^) is mainly produced via the metabolism of glutamine [38,39,43]. The degradation of glutamine to α-ketoglutarate in glutaminolysis can take place via two metabolic pathways: on the one hand, via the transamination reaction, and on the other hand, via the complete oxidation of glutamine catalyzed by the glutamine dehydrogenase (GDH). The amount of ammonium has a stronger impact on the culture than the lactate. However, it is difficult to determine an exact threshold for the ammonium concentration, since different critical values, between 2 and 10 mM, are given in the literature [39,40,44]. Glutamine is first converted into glutamate in glutaminolysis with ammonium splitting off in order to be introduced into the TCA as α-ketoglutarate, during which further ammonium is split off. Up to this point, 1 mol of glutamine has been converted into 2 mol of ammonium (see Figure 4).

As ammonium dissociates, ammonia is present as an uncharged molecule, which can diffuse freely through the cell membrane. As soon as a proton is released into the culture medium, the pH value in the medium decreases. In the cell, on the other hand, the pH value is increased by the uptake of a proton to form ammonium, which results in a pH gradient between the intracellular and extracellular space. In order to compensate for this gradient, the cell has to re-establish the pH-value under energy consumption by means of ion pumps. As a result, the basic energy requirement for maintaining the organism increases significantly and can therefore no longer be used for the proliferation or production of the antibody [36]. As a consequence, ammonium has a negative effect on cell growth and viability [38,39,40,43].

The concentration of inhibiting metabolites, such as lactate and ammonium, has to be monitored and preferably controlled for the above-mentioned reasons.

In this approach, in-situ turbidity and ex-situ Raman spectroscopy measurements are combined with an offline macroscopic Monod kinetic model in order to estimate substrate and metabolite concentrations (i.e., glucose and its metabolite lactate) as proof of principle. Macroscopic models lead to a more detailed process understanding and, in combination with experimental data, these models can be employed for the online prediction of substrates or metabolites. The connection between online cell concentration measurement and reliable online substrate or metabolite prediction will be one important step towards the online monitoring and controlling of bioprocesses.

## 2. Materials and Methods 

Chinese hamster ovary cells (CHO DG44) were used in bioreactor (Biostat^®^ B, Sartorius Stedim Biotech GmbH, Göttingen, Germany) cultivations for the production of a monoclonal antibody in a serum-free medium. The cultivation conditions were 36.8 °C, 5.0% carbon dioxide, 433 rpm, pH 7.1 and 60.0% dissolved oxygen (pO_2_). The in-situ turbidity probe (transmission, 880 nm, HiTec Zang GmbH, Herzogenrath, Germany) was connected to a LabManager^®^ (HiTec Zang GmbH). Raman spectroscopy was performed using an ex-situ Raman probe (Diode laser, 785 nm, Ocean Optics B.V, Ostfildern, Germany) in order to correlate the spectral data and the viable cell concentration using the NIPALS algorithm derived from the software The Unscrambler^®^ (CAMO Software AS., Oslo, Norway).

Offline viable and total cell concentration were repeatedly determined by using a Neubauer chamber (BRAND GMBH + CO KG, Wertheim, Germany), microscope (Motic BA 310, Motic Deutschland GmbH, Wetzlar, Germany), and trypan blue solution (0.4%, Sigma-Aldrich, St. Louis, MO, USA) as dye for the detection of dead cells. Glucose and lactate were repeatedly measured using a LaboTRACE compact (TRACE Analytics GmbH, Braunschweig, Germany). The monoclonal antibody was quantified by Protein A chromatography (PA ID Sensor Cartridge, Applied Biosystems, Bedford, MA, USA). Dulbecco’s PBS buffer (Sigma-Aldrich, St. Louis, MO, USA) was used as loading buffer at pH 7.4 and as elution buffer at pH 2.6. The absorbance was monitored at 280 nm.

The macroscopic kinetic model was developed in Aspen Custom Modeler V8.4 (Aspen Technology, Inc., Bedford, MA, USA). Since the cell cultures were performed in fed-batch mode with daily bolus feed additions, the model equations were extended by feeding terms. Consequently, the bioreactor volume was considered as well.

## 3. Results

### 3.1. Online Cell Concentration Measurement

**Figure 5:**
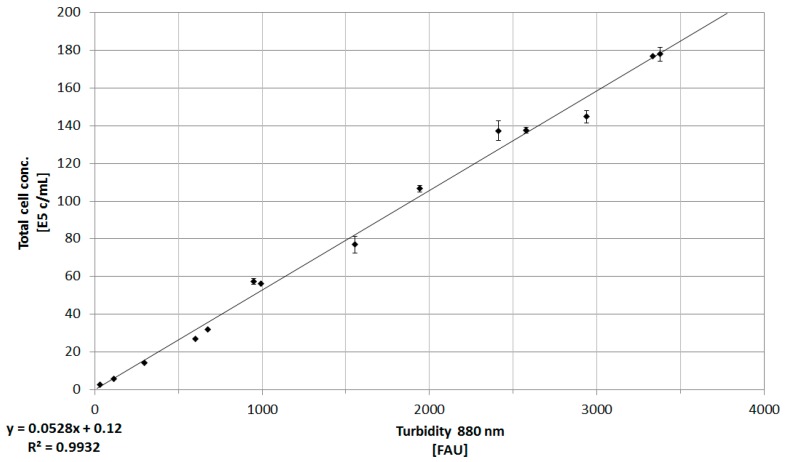
Correlation between offline determination of total cell concentration (E5 cells/mL) and online turbidity measurements (880 nm, FAU). Error bars represent the double determination of cell concentration, as well as the error of the probe (0.75%). The coefficient of determination for this linear relation is >0.99.

The basic principle of the correlation between process, state, and target variables is shown in Figure 1. This relationship, alongside the results from online turbidity and Raman measurements, as well as macroscopic modeling, is shown and discussed in detail in the following. The in-situ measurement of the turbidity of the cell culture suspension is one of the most applied techniques for biomass monitoring, due to the simplicity of this method [2]. Here, an online transmission probe using a wavelength of 880 nm was implemented in order to record the total cell concentration of two CHO DG44 bioreactor cultivations. The correlation between the turbidity measurements (formazine attenuation units, FAU) and total cell concentration (E5 cells/mL) is shown as an example for one bioreactor cultivation in Figure 5. The linear relation was found to be in good agreement for two bioreactor cultivations (R^2^ ≥ 0.97 ± 0.02).

Compared to the determination of the total cell concentration using turbidity probes, Raman spectroscopy combined with chemometric techniques can be used to predict substrate concentration, i.e., glutamine or glutamic acid, or viable and total cell concentration [2]. Using this approach, ex-situ Raman spectroscopy and PLS were used in order estimate the viable cell concentration offline. The PLS model was established using the NIPALS algorithm alongside cross-validation. Four factors are needed in order to explain 94% of the response variance. The resulting response prediction generates a sufficient model error (RMSE ≤ 13.82, R^2^ ≥ 0.92) for estimating the experimental viable cell concentration.

**Figure 6:**
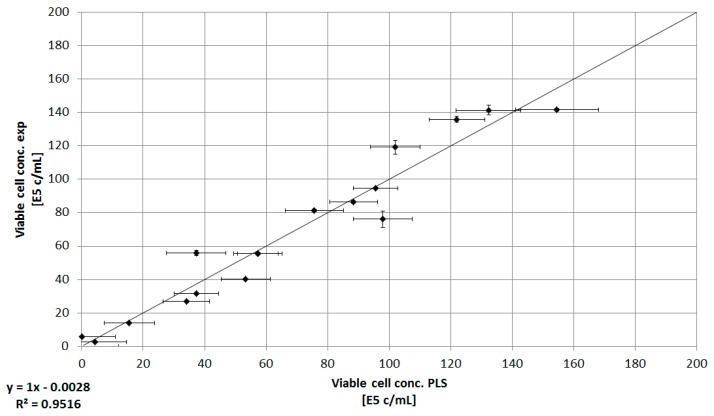
Correlation between offline viable cell concentration (E5 cells/mL) determination and PLS (NIPALS)-based viable cell concentration (E5 cells/mL). Error bars represent the double determination of cell concentration, as well as the model error (RMSE ≤ 13.82). The coefficient of determination for this linear relation is >0.95.

The correlation between the predicted viable cell concentration that results from the NIPALS algorithm and the experimental viable cell concentration is shown in Figure 6. Considering the nature of ex-situ spectral data acquisition (e.g., possible long hold up, variations of process variables in sampling loop), this prediction method seems to be quite sufficient for determining the viable cell concentration in the bioreactor.

**Figure 7:**
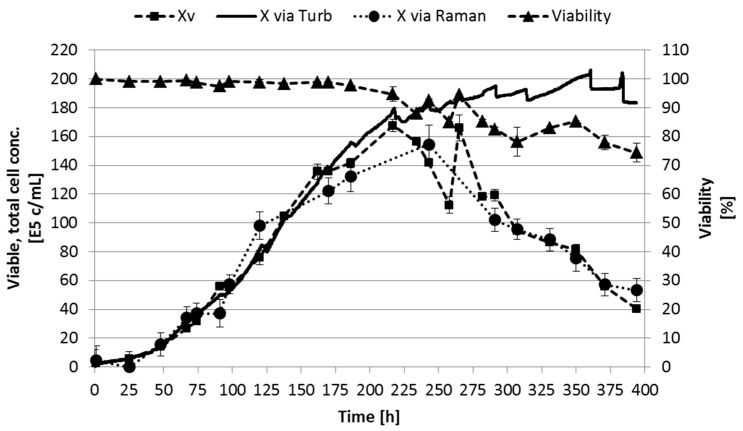
Viable and total cell concentration based on Raman spectroscopy coupled to PLS and turbidity, respectively, compared to experimental viable cell concentration. Error bars represent the double determination of cell concentration and viability, as well as the model error of PLS (RMSE ≤ 13.82).

Both techniques generate reproducible data and predictions. The acquisition of turbidity is more easily available and is simpler to integrate and interpret than Raman spectroscopy coupled to PLS. However, turbidity probes are only able to determine the total cell concentration based on the optical density of the culture suspension instead of the viable concentration and are affected by air bubbles and noncellular particles [2]. Regarding the productivity of secondary metabolites or recombinant proteins like monoclonal antibodies in the stationary phase of a (fed-)batch culture, the cellular state of the culture, and therefore its viability, is of utmost importance [45]. Hence, techniques that are able to determine the viable cell concentration are in favor. This prediction can efficiently be accomplished by Raman spectroscopy or, for example, near-infrared and impedance measurements [2]. The discrepancy between the results of Raman spectroscopy and turbidity can be seen in Figure 7.

**Figure 8:**
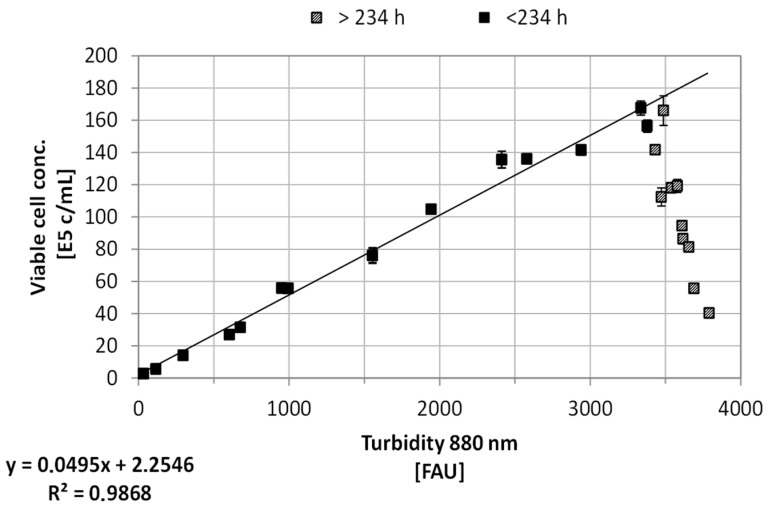
Correlation between offline viable cell concentration (E5 cells/mL) determination and online turbidity measurements (880 nm, FAU) prior to and after 234 h bioreactor cultivation. Error bars represent the double determination of cell concentration, as well as the deviation of the probe (0.75%). The coefficient of determination for this linear relation is >0.98 for *t* < 234 h.

The difference between the results of turbidity (total cell concentration) and Raman (viable cell concentration) measurements is expected. As can be seen in Figure 7 and Figure 8, the turbidity is in good correlation to the viable cell concentration due to the high viability of the cell culture. However, after approximately 234 h culture time, the turbidity probe measurements do not correlate to the viable cell concentration any more. Here, the PLS model is able to predict the decreasing viable cell concentration. The slowly rising cell concentration based on turbidity after 234 h is mainly due to cell accumulation in the probe gap. After the probe positioning in the bioreactor was adjusted, this increase did not occur again.

In conclusion, turbidity data can efficiently be used as process variable for process control if the cellular viability does not significantly differ (≥10%, see Figure 7, ≥234 h) between viable and total cell concentration. Besides, according to the more complex interpretation of Raman spectra, this technique is able to predict the cellular state more reliable. Methods are available that can acquire spectra and integrate them into PLS models, resulting in the online determination of viable cell, substrate, and metabolite concentrations [46,47]. For this, PLS models have to be robust and validated using validation data sets. By increasing the range of these validation data sets it is possible to reduce the model error significantly [48].

### 3.2. Macroscopic Kinetic Modeling

Macroscopic kinetic models are being used in order to simulate the cellular state and productivity in-silico [23,24,29,49]. In this approach, a Monod type model is used for the offline prediction of glucose concentration. This methodology will be enhanced in future experimental set-ups to predict the substrate concentration online and implement a control strategy utilizing an OPC network. The model consists of Monod-type equations that describe the time-dependent state of the viable cell [X_V_], glucose [GLC], glutamine [GLN], lactate [LAC], ammonium [AMM], and monoclonal antibody [mAb] concentration. Model parameters such as Monod constants (K_glc_, K_gln_, K_Ilac_, K_Iamm_), µ_max_, and yield coefficients (Y_X/glc_, Y_X/glc_, Y_X/glc_) are calculated based on bioreactor cultivations or are adopted from literature. The Equations (1) to (9) are given in the following and are mostly adopted from Xing et al. [49]. The employed model parameters are given in the following Table 2.

(1) $$\frac{d[X_{V}]}{\mathrm{dt}}=\left(\mu -\mu_{d}\right)\cdot [X_{V}],$$

(2) $$\mu =\mu_{\max}\cdot \frac{\left[\mathrm{GLC}\right]}{K_{\mathrm{glc}}\;+\;\left[\mathrm{GLC}\right]}\cdot \frac{\left[\mathrm{GLN}\right]}{K_{\mathrm{gln}}\;+\;\left[\mathrm{GLN}\right]}\cdot \frac{K_{\mathrm{Ilac}}}{K_{\mathrm{Ilac}}\;+\;\left[\mathrm{LAC}\right]}\cdot \frac{K_{\mathrm{Iamm}}}{K_{\mathrm{Iamm}\;}+\;\left[\mathrm{AMM}\right]},$$

(3) $$\mu_{d}=k_{d}\cdot \frac{\left[\mathrm{LAC}\right]}{K_{\mathrm{Dlac}}\;+\;\left[\mathrm{LAC}\right]}\cdot \frac{\left[\mathrm{AMM}\right]}{K_{\mathrm{Damm}}\;+\;\left[\mathrm{AMM}\right]},$$

(4) $$\frac{d\left[\mathrm{GLC}\right]}{\mathrm{dt}}=-\left(\frac{\mu \;-\mu_{d}}{Y_{\frac{X}{\mathrm{GLC}}}}+m_{\mathrm{glc}}\right)\cdot [X_{V}],$$

(5) $$\frac{d\left[\mathrm{GLN}\right]}{\mathrm{dt}}=\;-\left(\frac{\mu \;-{\;\mu }_{d}}{Y_{\frac{X}{\mathrm{GLN}}}}+m_{\mathrm{gln}}\right)\cdot \left[X_{V}\right],$$

(6) $$m_{\mathrm{gln}}=\frac{a_{1}\cdot \left[\mathrm{GLN}\right]}{a_{2\;}+\;\left[\mathrm{GLN}\right]},$$

(7) $$\frac{d\left[\mathrm{LAC}\right]}{\mathrm{dt}}=Y_{\frac{\mathrm{LAC}}{\mathrm{GLC}}}\cdot \;\left(\frac{\mu \;-{\;\mu }_{d}}{Y_{\frac{X}{\mathrm{GLC}}}}+m_{\mathrm{glc}}\right)\cdot [X_{V}],$$

(8) $$\frac{d\left[\mathrm{AMM}\right]}{\mathrm{dt}}=Y_{\frac{\mathrm{AMM}}{\mathrm{GLN}}}\cdot \;\left(\frac{\mu \;-{\;\mu }_{d}}{Y_{\frac{X}{\mathrm{GLC}}}}\right)\cdot [X_{V}]-r_{\mathrm{Amm}}\cdot [X_{V}],$$

(9) $$\frac{d\left[\mathrm{mAb}\right]}{\mathrm{dt}}=Q_{\mathrm{mAb}}\cdot [X_{V}]\cdot \left(1-\frac{\mu }{\mu_{\max\;}}\right),$$

**Figure 9:**
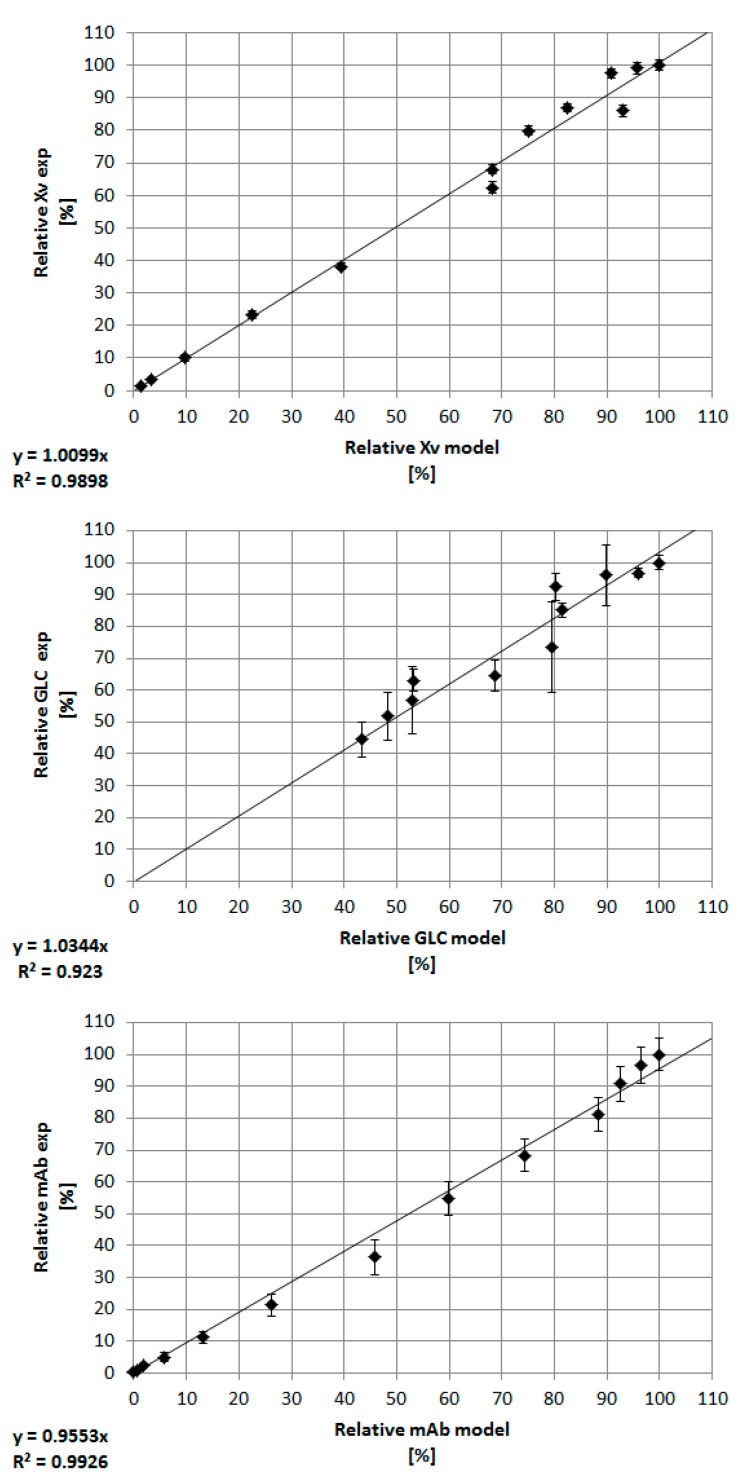
Correlation between experimental and predicted values for the viable cell, product, and glucose concentration. The error bars represent the standard deviation of various bioreactor cultivations. The coefficient of determination is ≥0.92.

**Figure 10:**
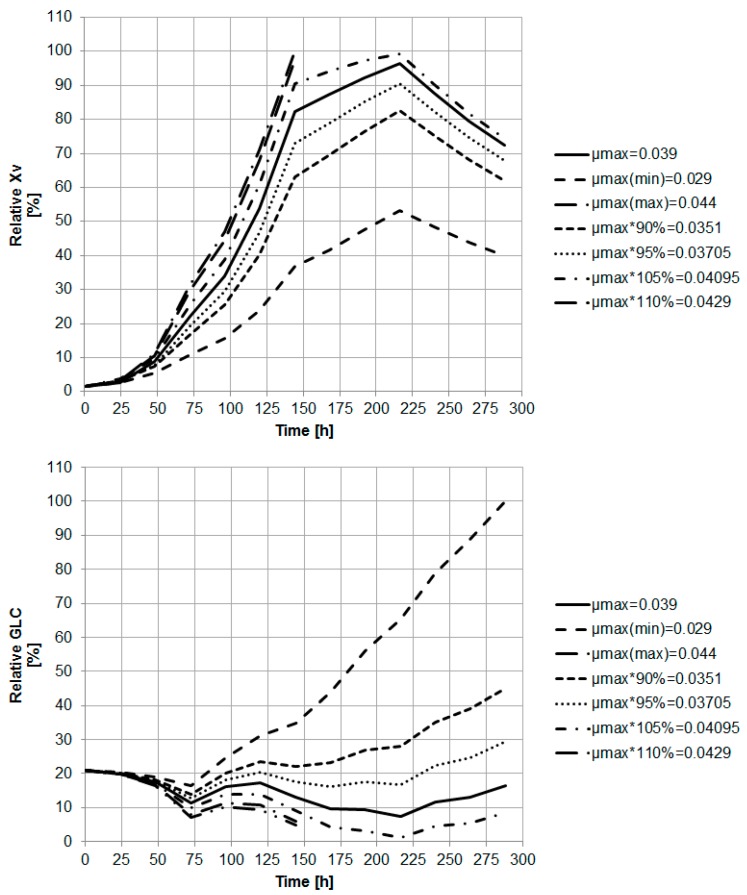
Influence of µ_max_ on the viable cell and glucose concentration. µ_max_ values represent data reported for mammalian cells (min, max), as well as ±5% and ±10% of the experimental determined value.

The cellular state, together with the product and glucose concentration, can be predicted sufficiently, as seen in Figure 9. The coefficient of determination for each prediction is greater than 0.92. In order to improve the prediction quality, the Markov Chain Monte Carlo method can be used for the prediction of parameters that are difficult to obtain such as m_glc_, K_gln_, or K_glc_ [49]. However, even parameters that can be derived from experimental data [23], such as µ_max_ or yield coefficients (e.g., Y_X/glc_, Y_X/gln_), exhibit a significant influence on model variables, especially [X_v_] and [GLC]. The model parameter µ_max_, for example, varied according to the minimal and maximum values reported for mammalian cells, as well as ±5% and ±10% of the experimentally determined value in order to examine its influence on the course of the viable cell and glucose concentration (Figure 10).

As can be seen in Equations (1)–(9) and Figure 10, the maximum growth rate µ_max_ may be the key parameter of this macroscopic model. An increase of the maximum growth rate has significant impact on growth and maximum cell concentration, and consequently on substrate concentration. Accompanied by the increased cell concentration, the glucose concentration decreases more rapidly (constant feed strategy assumed). This relation is due to the dependency of the substrate concentration on the cell concentration (Equation (4)), which therefore affects the temporal growth rate (Equation (1)) and hence the maximum growth rate µ_max_ (Equation (2)). This effect is not surprising, but clearly shows the importance of an exact parameter determination methodology for highly sensitive parameters. Here, µ_max_ exhibited the most significant influence on the course of model variables. For example, online measurements such as turbidity can efficiently be used for the determination of µ_max_, due to their very high sample resolution. The improved data sets result in a significantly improved data consistency and in a more precise model parameter prediction.

### 3.3. Combination of Experimental Data and Predictive Modeling

**Figure 11:**
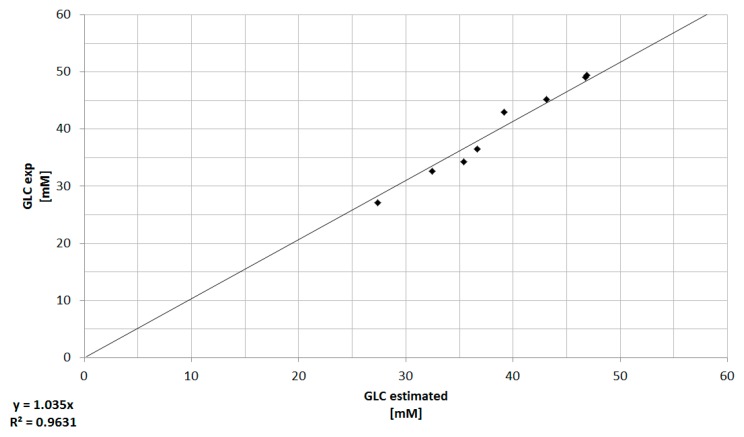
Comparison between offline measurements (exp) and model-based prediction of the glucose concentration. The deviation represents the offline double determination of the glucose concentration. The coefficient of determination for this relation is ≥0.96.

The prediction of the experimental viable cell and substrate concentration using a macroscopic kinetic model shows a sufficient agreement. In order to integrate this Monod type model into the process environment, online turbidity data, which represents the cell concentration, were connected to the model for the prediction of glucose concentration (Equation (4)). In future approaches, this shall lead to a simple method for the approximate determination of growth limiting substrates, such as glucose. The comparison between offline measurements and model-based prediction of the glucose concentration can be seen in the following Figure 11.

**Figure 12:**
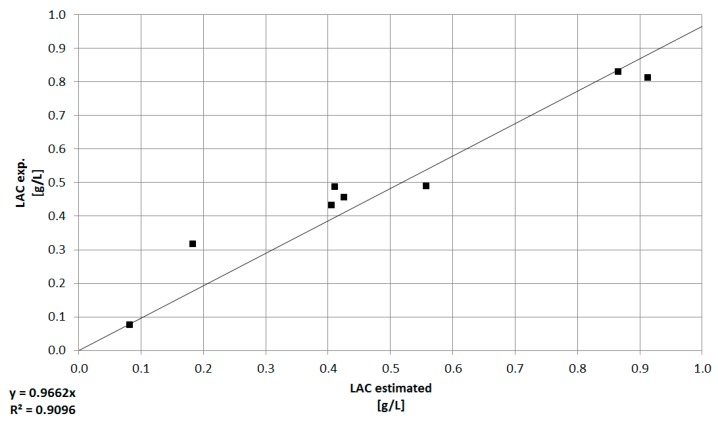
Comparison between offline measurements (exp) and model-based prediction of the lactate concentration. The deviation represents the offline double determination of the lactate concentration. The coefficient of determination for this relation is ≥0.90.

As it can be seen in Figure 11, the model based determination of the glucose concentration sufficiently (R^2^ ≥ 0.96) predicts the offline measured values, especially in the pre-feed phase (≤67 h) of the cultivation. The prediction of the glucose concentration in the feed phase is significantly more challenging, since physiological effects of the feed media on the cellular state (e.g., substrate limitation/inhibition, change from cell growth to production, influence of feed media components) are not described by this model at this time. Furthermore, any process disturbances result in a non-ideality of the cellular state or substrate concentration and are difficult to predict using macroscopic models. In addition, the viable cell concentration determination based on turbidity measurements is dependent on a viable cell state (Viability ≥90%). The discrepancy between experimentally determined viable cell concentration and turbidity-based calculation (Figure 8) results in an additional deviation of the estimated glucose concentration from the available glucose in culture. Besides the need for a reliable estimation or even prediction of the substrate concentration, metabolites have to be predicted, because lactate or ammonium are responsible for inhibiting cell proliferation (see Figure 3 and Figure 4). The comparison between offline measurements and model-based prediction of the lactate concentration can be seen in the following Figure 12.

Nevertheless, turbidity measurements combined with a macroscopic kinetic model can be used for an online estimation of substrate concentration and, in future approaches, will be validated and extended to metabolites (e.g., ammonium), since they play a significant role in cell dynamics and protein production as described above (see Figure 3 and Figure 4).

## 4. Conclusions

The presented approach of online turbidity and Raman spectroscopy depicts their advantages and disadvantages regarding their implementation in bioprocesses. Turbidity measurements seem to be more suited to a fast and simple technique for interpreting cellular state monitoring, despite the limitations on total cell concentration. Therefore, spectroscopy methods that display the cellular viability, such as Raman coupled to chemometric analysis (e.g., PLS), are in favor. However, the interpretation of the acquired spectra can be complex and are not always obvious. The correlation of total and viable cell concentration using online turbidity (R^2^ ≥ 0.99) and Raman spectroscopy (R^2^ ≥ 0.95), respectively, is sufficient and capable of approximately predicting the glucose (R^2^ ≥ 0.96) and lactate concentration (R^2^ ≥ 0.90) by employing a macroscopic Monod-type model.

In order to measure, analyze, monitor, and ultimately control all important attributes of a bioprocess, reliable measurement techniques are essential. In future approaches, the prediction of the glucose concentration will be further validated and implemented into a control strategy using turbidity or Raman spectroscopy, as well as an OPC platform. This strategy will merge online measurements, macroscopic models, and finally advanced process control for robust and reliable batch or continuous operation.

Those steps are also crucial for any innovation and optimization of the manufacturing technology of biologics such as, e.g., the transfer form batch to continuous processes. In a continuous operation, process robustness is even more crucial in order to prevent failures or instabilities, as the annual costs would be significantly higher than in a comparable batch operation. First studies on continuous biomanufacturing prove better reproducibility [51]. Appropriate process control strategies based on sound PAT tools, in combination with process modeling technologies, as shown here, will keep critical product quality attributes reliably within specification by controlling operation parameters within their design space.

Improved reliability in process operation will enable manufacturing optimization projects and even innovation in industrialization towards more economic manufacturing of innovative medicines for the patients’ sake [52,53,54,55,56,57].

## Figures and Tables

**Table 1:** Exemplary overview of methods that are able to measure process variables of mammalian cell culture quantitatively or qualitatively, according to [4,13]. UV/Vis, ultraviolet and visible spectroscopy; NIR, near-infrared spectroscopy; MIR mid-infrared spectroscopy; VCD, viable cell density.

Method	Component	Quantitative or Qualitative
UV/Vis	Cell density	Quantitative
Fluorescence	VCD, titer, tyrosine, tryptophan	Quantitative
Raman	Glc, Lac, Gln, Glu, Amm, VCD	Quantitative
NIR	Glc, Lac, Biomass, Gln, AMM, titer, viscosity	Quantitative and qualitative
MIR	Glc, EtOH, organic acids	Quantitative

**Table 2:** Model parameters used in the macroscopic Monod model for the simulation of CHO cultivations based on literature (Lit.) or experimental data (exp). Literature data were determined using fed-batch CHO cell culture and the markov chain monte carlo method for parameter estimation [49] or by the differential evolution technique [50].

Parameter	Description	Value	Unit	Source
µ_max_	Maximum growth rate	0.039	h^−1^	exp
k_d_	Maximum death rate	0.004	h^−1^	exp
K_glc_	Monod constant glucose	1.00	mM	[50]
K_gln_	Monod constant glutamine	0.047	mM	[49]
K_Ilac_	Monod constant lactate for inhibition	43.00	mM	[49]
K_Iamm_	Monod constant ammonium for inhibition	6.51	mM	[49]
K_Dlac_	Monod constant lactate for death	45.8	mM	[49]
K_Damm_	Monod constant ammonium for death	6.51	mM	[49]
Y_X/glc_	Yield coefficient cell conc./glucose	0.357	E9 cells mmol^−1^	exp
Y_X/gln_	Yield coefficient cell conc./glutamine	0.974	E9 cells mmol^−1^	[49]
Y_lac/glc_	Yield coefficient lactate/glucose	0.70	mmol mmol^−1^	exp
Y_amm/gln_	Yield coefficient ammonium/glutamine	0.67	mmol mmol^−1^	[49]
r_amm_	Ammonium removal rate	6.3	E-12 mmol cell^−1^·h^−1^	[49]
m_glc_	Glucose maintenance coefficient	69.2	E-12 mmol cell^−1^·h^−1^	[49]
a_1_	Coefficient for m_gln_	3.2	E-12 mmol cell^−1^·h^−1^	[49]
a_2_	Coefficient for m_gln_	2.1	mM	[49]
Q_mAb_	Specific production rate	1.51	E-12 g·c^−1^·h^−1^	exp

## References

[1] Hinz D.C. (2006). Process analytical technologies in the pharmaceutical industry: The FDA’s PAT initiative. Anal. Bioanal. Chem..

[2] Biechele P., Busse C., Solle D., Scheper T., Reardon K. (2015). Sensor systems for bioprocess monitoring. Eng. Life Sci..

[3] Food and Drug Administration (2004). Guidance for Industry. PAT—A Framework for Innovative Pharmaceutical Development, Manufacturing, and Quality Assurance. https://www.fda.gov/downloads/drugs/guidances/ucm070305.pdf.

[4] Musmann C., Joeris K., Markert S., Solle D., Scheper T. (2016). Spectroscopic methods and their applicability for high-throughput characterization of mammalian cell cultures in automated cell culture systems. Eng. Life Sci..

[5] Zobel-Roos S., Mouellef M., Siemers C., Strube J. (2017). Process Analytical Approach towards Quality Controlled Process Automation for the Downstream of Protein Mixtures by Inline Concentration Measurements Based on Ultraviolet/Visible Light (UV/VIS) Spectral Analysis. Antibodies.

[6] Bechmann J., Rudolph F., Gebert L., Schaub J., Greulich B., Dieterle M., Bradl H. (2015). Process parameters impacting product quality. BMC Proc..

[7] Alt N., Zhang T.Y., Motchnik P., Taticek R., Quarmby V., Schlothauer T., Beck H., Emrich T., Harris R.J. (2016). Determination of critical quality attributes for monoclonal antibodies using quality by design principles. Biologicals.

[8] Del Val I.J., Kontoravdi C., Nagy J.M. (2010). Towards the implementation of quality by design to the production of therapeutic monoclonal antibodies with desired glycosylation patterns. Biotechnol. Prog..

[9] Lourenço N.D., Lopes J.A., Almeida C.F., Sarraguça M.C., Pinheiro H.M. (2012). Bioreactor monitoring with spectroscopy and chemometrics: A review. Anal. Bioanal. Chem..

[10] Arnold S.A., Crowley J., Woods N., Harvey L.M., McNeil B. (2003). In-situ near infrared spectroscopy to monitor key analytes in mammalian cell cultivation. Biotechnol. Bioeng..

[11] Rhiel M., Ducommun P., Bolzonella I., Marison I., von Stockar U. (2002). Real-time in situ monitoring of freely suspended and immobilized cell cultures based on mid-infrared spectroscopic measurements. Biotechnol. Bioeng..

[12] Berry B., Moretto J., Matthews T., Smelko J., Wiltberger K. (2015). Cross-scale predictive modeling of CHO cell culture growth and metabolites using Raman spectroscopy and multivariate analysis. Biotechnol. Prog..

[13] Teixeira A.P., Oliveira R., Alves P.M., Carrondo M.J.T. (2009). Advances in on-line monitoring and control of mammalian cell cultures: Supporting the PAT initiative. Biotechnol. Adv..

[14] Otto M. (2017). Chemometrics. Statistics and Computer Application in Analytical Chemistry.

[15] Faassen S.M., Hitzmann B. (2015). Fluorescence spectroscopy and chemometric modeling for bioprocess monitoring. Sensors.

[16] Kroll P., Stelzer I.V., Herwig C. (2017). Soft sensor for monitoring biomass subpopulations in mammalian cell culture processes. Biotechnol. Lett..

[17] Hausmann R., Henkel M., Hecker F., Hitzmann B. (2017). Present Status of Automation for Industrial Bioprocesses. Current Developments in Biotechnology and Bioengineering.

[18] Pörtner R., Platas Barradas O., Frahm B., Hass V.C. (2017). Advanced Process and Control Strategies for Bioreactors. Current Developments in Biotechnology and Bioengineering.

[19] FDA, CDER, CBER, FDA, USDHHS (2009). Pharmaceutical Development Q8(R2). https://www.ich.org/fileadmin/Public_Web_Site/ICH_Products/Guidelines/Quality/Q8_R1/Step4/Q8_R2_Guideline.pdf.

[20] Esmonde-White K.A., Cuellar M., Uerpmann C., Lenain B., Lewis I.R. (2017). Raman spectroscopy as a process analytical technology for pharmaceutical manufacturing and bioprocessing. Anal. Bioanal. Chem..

[21] Luttmann R., Bracewell D.G., Cornelissen G., Gernaey K.V., Glassey J., Hass V.C., Kaiser C., Preusse C., Striedner G., Mandenius C.-F. (2012). Soft sensors in bioprocessing: A status report and recommendations. Biotechnol. J..

[22] Präve P. (1994). Handbuch der Biotechnologie. Mit 150 Tabellen, 26 Fließschemata sowie 80 Strukturformeln und Zahlreichen Weiteren Zusammenstellungen und Formeln.

[23] Yahia B.B., Malphettes L., Heinzle E. (2015). Macroscopic modeling of mammalian cell growth and metabolism. Appl. Microbiol. Biotechnol..

[24] Yahia B.B., Gourevitch B., Malphettes L., Heinzle E. (2016). Segmented linear modelling of CHO fed-batch culture and its application to large scale production. Biotechnol. Bioeng..

[25] Goudar C.T. (2012). Computer programs for modeling mammalian cell batch and fed-batch cultures using logistic equations. Cytotechnology.

[26] Goudar C.T., Konstantinov K.B., Piret J.M. (2009). Robust parameter estimation during logistic modeling of batch and fed-batch culture kinetics. Biotechnol. Prog..

[27] Goudar C.T., Joeris K., Konstantinov K.B., Piret J.M. (2005). Logistic equations effectively model mammalian cell batch and fed-batch kinetics by logically constraining the fit. Biotechnol. Prog..

[28] Jang J.D., Barford J.P. (2000). An unstructured kinetic model of macromolecular metabolism in batch and fed-batch cultures of hybridoma cells producing monoclonal antibody. Biochem. Eng. J..

[29] Sidoli F.R., Mantalaris A., Asprey S.P. (2004). Modelling of Mammalian Cells and Cell Culture Processes. Cytotechnology.

[30] Galleguillos S.N., Ruckerbauer D., Gerstl M.P., Borth N., Hanscho M., Zanghellini J. (2017). What can mathematical modelling say about CHO metabolism and protein glycosylation?. Comput. Struct. Biotechnol. J..

[31] Huang Z., Lee D.-Y., Yoon S. (2017). Quantitative intracellular flux modeling and applications in biotherapeutic development and production using CHO cell cultures. Biotechnol. Bioeng..

[32] Christen P., Jaussi R., Benoit R. (2016). Biochemie und Molekularbiologie. Eine Einführung in 40 Lerneinheiten.

[33] Schaub J., Clemens C., Schorn P., Hildebrandt T., Rust W., Mennerich D., Kaufmann H., Schulz T.W. (2010). CHO gene expression profiling in biopharmaceutical process analysis and design. Biotechnol. Bioeng..

[34] Konakovsky V., Clemens C., Müller M.M., Bechmann J., Berger M., Schlatter S., Herwig C. (2016). Metabolic Control in Mammalian Fed-Batch Cell Cultures for Reduced Lactic Acid Accumulation and Improved Process Robustness. Bioengineering.

[35] Schaub J., Clemens C., Kaufmann H., Schulz T.W. (2012). Advancing biopharmaceutical process development by system-level data analysis and integration of omics data. Adv. Biochem. Eng. Biotechnol..

[36] Glacken M.W. (1988). Catabolic Control of Mammalian Cell Culture. Nat. Biotechnol..

[37] Ozturk S.S., Hu W.-S. (2006). Cell Culture Technology for Pharmaceutical and Cell-Based Therapies.

[38] Newsholme P., Procopio J., Lima M.M.R., Pithon-Curi T.C., Curi R. (2003). Glutamine and glutamate—Their central role in cell metabolism and function. Cell Biochem. Funct..

[39] Andersen D.C., Goochee C.F. (1995). The effect of ammonia on the O-linked glycosylation of granulocyte colony-stimulating factor produced by Chinese hamster ovary cells. Biotechnol. Bioeng..

[40] Ozturk S.S., Riley M.R., Palsson B.O. (1992). Effects of ammonia and lactate on hybridoma growth, metabolism, and antibody production. Biotechnol. Bioeng..

[41] Zhou M., Crawford Y., Ng D., Tung J., Pynn A.F.J., Meier A., Yuk I.H., Vijayasankaran N., Leach K., Joly J. (2011). Decreasing lactate level and increasing antibody production in Chinese Hamster Ovary cells (CHO) by reducing the expression of lactate dehydrogenase and pyruvate dehydrogenase kinases. J. Biotechnol..

[42] Zagari F., Jordan M., Stettler M., Broly H., Wurm F.M. (2013). Lactate metabolism shift in CHO cell culture: The role of mitochondrial oxidative activity. New Biotechnol..

[43] Hong J.K., Cho S.M., Yoon S.K. (2010). Substitution of glutamine by glutamate enhances production and galactosylation of recombinant IgG in Chinese hamster ovary cells. Appl. Microbiol. Biotechnol..

[44] Xing Z., Li Z., Chow V., Lee S.S. (2008). Identifying inhibitory threshold values of repressing metabolites in CHO cell culture using multivariate analysis methods. Biotechnol. Prog..

[45] Klein T., Heinzel N., Kroll P., Brunner M., Herwig C., Neutsch L. (2015). Quantification of cell lysis during CHO bioprocesses: Impact on cell count, growth kinetics and productivity. J. Biotechnol..

[46] Bhatia H., Mehdizadeh H., Drapeau D., Yoon S. (2018). In-line monitoring of amino acids in mammalian cell cultures using raman spectroscopy and multivariate chemometrics models. Eng. Life Sci..

[47] Buckley K., Ryder A.G. (2017). Applications of Raman Spectroscopy in Biopharmaceutical Manufacturing: A Short Review. Appl. Spectrosc..

[48] Zhao N., Wu Z.-S., Zhang Q., Shi X.-Y., Ma Q., Qiao Y.-J. (2015). Optimization of Parameter Selection for Partial Least Squares Model Development. Sci. Rep..

[49] Xing Z., Bishop N., Leister K., Li Z.J. (2010). Modeling kinetics of a large-scale fed-batch CHO cell culture by markov chain monte carlo method. Biotechnol. Prog..

[50] Craven S., Shirsat N., Whelan J., Glennon B. (2013). Process model comparison and transferability across bioreactor scales and modes of operation for a mammalian cell bioprocess. Biotechnol. Prog..

[51] Subramanian G. (2017). Continuous Biomanufacturing—Innovative Technologies and Methods.

[52] Gronemeyer P., Thiess H., Zobel S., Ditz R., Strube J., Subramanian G. (2017). Integration of Upstream and Downstream in Continuous Biomanufacturing. Continuous Biomanufacturing.

[53] Gronemeyer P., Ditz R., Strube J. (2014). Trends in Upstream and Downstream Process Development for Antibody Manufacturing. Bioengineering.

[54] Kornecki M., Mestmäcker F., Zobel-Roos S., Heikaus de Figueiredo L., Schlüter H., Strube J. (2017). Host Cell Proteins in Biologics Manufacturing: The Good, the Bad, and the Ugly. Antibodies.

[55] Zobel S., Helling C., Ditz R., Strube J. (2014). Design and operation of continuous countercurrent chromatography in biotechnological production. Ind. Eng. Chem. Res..

[56] Wurm F.M. (2004). Production of recombinant protein therapeutics in cultivated mammalian cells. Nat. Biotechnol..

[57] Sommerfeld S., Strube J. (2005). Challenges in biotechnology production—Generic processes and process optimization for monoclonal antibodies. Chem. Eng. Process. Process Intensif..

